# The role of coping strategies and psychological resilience in shaping academic stress levels

**DOI:** 10.1371/journal.pone.0360002

**Published:** 2026-10-07

**Authors:** Zuzanna Gurzyńska, Karolina Kucka, Marcin Trzciński, Katarzyna Topolska, Katarzyna Nowicka-Sauer, Krzysztof Sobczak

**Affiliations:** 1 Department of Sociology of Medicine and Medical Communication, Medical University of Gdansk, Gdansk, Poland; 2 Student Scientific Circle of Medical Communication, Medical University of Gdansk, Gdansk, Poland; 3 Faculty of Information and Communication Technology, Department of Control Systems and Mechatronics, Wroclaw University of Science and Technology, Wroclaw, Poland; 4 Faculty of Medicine, Division of Family Medicine, Medical University of Gdansk, Gdansk, Poland; 5 Department of Medical Education and Communication, Poznan University of Medical Sciences, Poznan, Poland; Alexandria University Faculty of Nursing, EGYPT

## Abstract

The period of university studies is associated with numerous stressors that can negatively affect the psychological functioning of young adults. This study aimed to investigate the relationship between perceived stress levels, preferred coping strategies, and psychological resilience. The analysis focused on resilience as a potential moderator of the relationship between coping strategies and stress intensity. The study included 607 Polish students. A significant part of the group was medical students (61.72%). The study employed the Perceived Stress Scale (PSS-10), the Coping Inventory (Mini-COPE), the Resilience Scale (SPP-25), and used an author-developed questionnaire to collect sociodemographic information. The respondents exhibited moderate stress intensity (M = 22.54; SD = 7.04) and a moderately high level of general resilience (M = 59.39; SD = 15.37). Among coping strategies, acceptance (M = 1.94) and active coping (M = 1.85) were chosen most frequently, while turning to religion and helplessness were chosen least frequently. Analysis of personal resources showed the highest scores in openness to experience and sense of humor. Interaction effects rarely reached statistical significance. The study group showed a profile of students who were stressed but had functional coping resources. The moderation analysis results indicate the complex nature of the relationships among the variables studied—although psychological resilience is a key resource, its role as a moderator of the relationship between coping strategies and stress is not universal and emerges only in specific areas of functioning. The data obtained suggest that psychological interventions should focus on strengthening existing resources (such as active coping) and developing resilience, which may serve as a buffer against the negative effects of academic stress.

## Introduction

Stress is a common phenomenon that significantly affects human functioning across various areas of life. In an academic context, stress is particularly evident among students who face intense cognitive, emotional, and social demands during their studies. In many cases, these demands may exceed students’ available adaptive resources. Research indicates that this results in a deterioration in quality of life [1]. Some researchers suggest that these conditions may also be linked to academic performance and the quality of social interactions [2].

Certain fields of study seem particularly prone to stress. This is related to the specific nature of education, including high academic demands, pressure of responsibility, and exposure to emotionally stressful situations [3,4]. So far, the results emphasize the need for in-depth analyses of academic stress that consider not only its intensity but also its consequences. According to the transactional model, stress occurs when a situation is assessed as exceeding an individual’s coping abilities [5].

The health consequences of chronic academic stress depend heavily on an individual’s coping styles and their psychological resilience to real or perceived stress [6]. Coping refers to the cognitive and behavioral efforts made to master, tolerate, or reduce external and internal demands and conflicts. Consequently, coping strategies are defined as specific actions, a series of actions, or thought processes used to manage a stressful or unpleasant situation or to modify one’s reaction to it. In the literature, these strategies are primarily classified as either adaptive or maladaptive. Adaptive coping strategies include both problem-focused and emotion-focused coping. Problem-focused coping typically centers on actively addressing the issue, which may involve seeking professional support such as counseling or cognitive behavioral therapy. Emotion-focused coping, on the other hand, includes strategies to manage emotional distress, such as practicing mindfulness, meditation, and yoga; using humor and jokes; engaging in spiritual or religious pursuits; engaging in physical activity or breathing exercises; and actively seeking social support. Conversely, maladaptive coping responses involve detrimental behaviors such as the avoidance of the stressful condition, withdrawal from a stressful environment, disengagement from stressful relationships, and the misuse of drugs and/or alcohol. Research indicates that adaptive strategies are associated with better mental functioning and long-term well-being, whereas maladaptive ones provide temporary relief but ultimately intensify the negative consequences of stress [7].

The way students cope with stress during their studies appears closely associated with students’ quality of life and with behaviors related to professional socialization and learning effectiveness, although this cross-sectional study does not allow firm conclusions about causal directions. Many students struggle with high stress levels, which may result in depression and burnout even before they start professional work [8,9]. Psychoeducational interventions for university students, grounded in research in this area, devote considerable attention to the role of personal resources in adaptation. One of these is psychological resilience, defined as the ability to maintain relatively stable functioning and effective adaptation in the face of adversity [10,11]. Research indicates that higher resilience is associated with better mental well-being and less susceptibility to the negative consequences of stress [12]. Resilience can therefore act as a buffering factor, modifying the relationship between coping strategies and stress levels.

From the perspective of the transactional model of stress [6], coping effectiveness depends not only on the strategies an individual adopts but also on the personal resources available during the appraisal and coping process. Lazarus and Folkman proposed that stress results from a dynamic transaction between environmental demands and an individual’s perceived ability to manage them, with coping outcomes being contingent on available psychological resources rather than determined solely by the coping strategy itself. Within this framework, psychological resilience may be conceptualized as an internal resource that promotes adaptive appraisal, emotional regulation, cognitive flexibility, and persistence when confronted with adversity. Consequently, resilience is expected not only to protect mental well-being directly but also to modify the strength of the association between coping strategies and perceived stress, functioning as a moderator of this relationship.

Although numerous studies have addressed stress, coping strategies, and resilience, significant gaps remain in understanding the nature of the relationships among these constructs [13,14]. Existing literature suggests that these associations are complex and not yet sufficiently clarified [15,16]. In particular, there is an ongoing debate concerning the conceptualization and operationalization of resilience and resiliency, as well as their role as moderating factors across various psychological processes [13,14]. Despite growing interest in these issues, there is still a lack of integrative analyses that simultaneously account for both mediating and moderating mechanisms underlying the interplay between stress and coping strategies. Addressing this gap is particularly important for developing more effective psychological interventions that strengthen individual resources and enhance adaptive coping [15,17]. Therefore, the present study examines the relationships among perceived stress, coping strategies, and psychological resilience, with a specific focus on resilience’s moderating role in the relationship between coping strategies and stress levels.

## Materials and methods

### Aim and objectives

This study aimed to analyze stress levels associated with students’ life circumstances. We reviewed the frequency of stress-coping strategies and measured psychological resilience as a moderator.

### Study design

The cross-sectional study used a quantitative method with the Computer-Assisted Web Interview (CAWI) technique. The digitized survey was posted on a professional e-research portal (www.eBadania.pl), which guarantees the full protection of the collected data. The website automatically blocks the IP address of any device that has already submitted survey responses, thereby reducing the risk of duplicate participation.

Participation was voluntary and anonymous. Inclusion criteria comprised being an active university student in Poland and providing informed consent to participate. Students self-reported their status, which we verified through questions about university affiliation and field of study. We applied no additional formal exclusion criteria beyond non-student status and incomplete questionnaires.

### Data collection

The data was collected from May 28, 2023, to December 31, 2023. We reached the study participants through mailings, university newsletters, research clubs, contact with student group leaders, and nationwide social media. We also used the administrative structures of individual universities, asking them to email us about the possibility of participating in the survey.

### Participants identification

The study participants were students from 46 universities across Poland. More than half of the students surveyed were studying at the Medical University of Gdansk when they completed the questionnaire (61.7%). Most respondents were women (77.4%). Students aged 18–21 accounted for over half of the respondents (55.2%). We collected 611 questionnaires. Before analysis, we assessed completeness. Questionnaires with substantial missing data (defined as missing responses in variables required for analysis) were excluded from further processing. In total, four questionnaires met this criterion and were removed. Ultimately, data from 607 questionnaires were included in the statistical analyses (Table 1). Of all respondents from all universities, 30.2% were medical students, 11.6% were pharmacy students, 6.8% were psychology students, and the remaining 51.4% were students of other fields. 97.5% of respondents studied one field, and 81.1% declared that it was their first choice. Among the respondents, 58.2% reported not combining their studies with work. 48.9% of respondents reported having a partner.

**Table 1:** Respondents’ sociodemographic data (N = 607).

Category	Variable	N	%
Sex	Women	470	77.4
	Men	118	19.4
	Other	15	2.5
	Prefer not to answer	4	0.7
Age	18-21	335	55.2
	≥22	272	44.8
University	Medical University of Gdansk	374	61.7
	Academy of Fine Arts in Gdańsk	86	14.2
	University of Gdańsk	41	6.8
	Gdańsk University of Technology	14	2.3
	University of Warmia and Mazury in Olsztyn	12	2.0
	Other	80	13.2
Study major	Medicine	183	30.3
	Pharmacy	70	11.6
	Psychology	41	6.8
	Nursing	35	5.8
	Physiotherapy	29	4.8
	Other	249	40.7
Year of study	1st	201	33.1
	2nd	170	28.1
	3rd	95	15.7
	4th	67	11.1
	5th	51	8.4
	6th	21	3.5
Mode of study	Full-time	541	89.1
	Part-time	53	8.7
	Both full-time and part-time	13	2.1
Number of majors	One	592	97.5
	Two or more	15	2.5
Combining studies with work	Yes	254	41.8
	No	353	58.2
Having a relationship	Yes	297	48.9
	No	310	51.1
Main field of study as first choice	Yes	492	81.1
	No	115	18.9

### Ethical considerations

The study design was approved by the Independent Bioethics Committee for Scientific Research at the Medical University of Gdansk (NKBBN/245/2023). Before participating, all respondents were informed about the study’s nature and purpose. All participants provided electronic informed consent before beginning the questionnaire. Participants indicated their consent by selecting the appropriate option in the online survey form before accessing the questionnaire. Participation was voluntary, and respondents could withdraw from the study. The study was anonymous, and no sensitive personal data was collected.

### Measures

The study used the Perceived Stress Scale (PSS-10) in its Polish adaptation [18]. The scale consists of 10 questions concerning thoughts and feelings related to events experienced in the last month, using a 5-point Likert scale. We performed the analysis by calculating the overall score and using a sten scale. Higher PSS-10 scores indicate greater stress intensity. The scale’s internal consistency is indicated by a Cronbach’s alpha of 0.86.

To measure coping strategies, we used the Polish version of the Mini-COPE Inventory [19]. The questionnaire contains 30 statements about ways of coping in a very difficult situation, to which respondents respond using a 4-point Likert scale. The split-half reliability of the Polish version of the Mini-COPE inventory obtained a Guttman index of 0.87. Statistical analyses were performed in accordance with the recommendations for standardizing the Polish version of the scale, using seven subscales: active coping, helplessness, support seeking, avoidance, turning to religion, acceptance, and sense of humor.

The Resilience Measurement Scale (SPP-25) consists of 25 statements [11]. Respondents rate each statement on a 5-point Likert scale. The scale results are an overall score for resilience and scores for five factors: Perseverance and determination in action (α = 0.72), Openness to new experiences and sense of humor (α = 0.68), Personal competences to cope and tolerance for negative emotions (α = 0.74), Tolerance of failure and treating life as a challenge (α = 0.67), Optimistic outlook on life and the ability to mobilize in difficult situations (α = 0.75). The higher the number of points obtained, the higher the level of resilience. The STEN scale results are interpreted as follows: 1–4 indicate low resilience, 5–6 indicate average resilience, and 7–10 indicate high resilience. The entire scale has an internal consistency of Cronbach’s alpha = 0.89. An author-developed questionnaire was used to collect socio-demographic data, including gender, age, year of study, university affiliation, and field of study.

### Statistical analysis

The study applied statistical methods appropriate for analyzing ordinal questionnaire data and testing complex relationships between variables, and all analyses were conducted using IBM SPSS Statistics 26. In the first stage, descriptive statistics were calculated, including means, standard deviations, medians, ranges, skewness, and kurtosis, to characterize the sample and score distributions on the PSS-10, Mini-COPE, and SPP-25 scales. Next, we assessed the reliability of the measurement instruments using Cronbach’s alpha coefficients and the Guttman split-half coefficient for the Mini-COPE inventory. Spearman’s rank-order correlations were used to examine the strength and direction of relationships between perceived stress, coping strategies, and psychological resilience. The lack of normality of the variables analyzed justified the choice of this nonparametric method. We then conducted a moderation analysis using linear regression. In these models, coping strategies were included as predictors, stress level as the dependent variable, and components of psychological resilience as moderators. Moderation effects were tested using interaction terms of the type of coping strategy × resilience resource. The analyses reported R and R² coefficients, changes in R², and the statistical significance of interaction effects. All statistical tests were conducted using an assumed significance level of p = 0.05. For comparisons between medical and non-medical students, we also calculated effect sizes: Cohen’s d for independent-samples t-tests and the rank-biserial correlation coefficient r for Mann–Whitney U tests. Because 14 comparisons were performed, the Bonferroni-adjusted significance threshold was set at α = 0.05/14 = 0.0036. The invariance of the moderation models across the two subgroups was verified with a Chow-type test of coefficient equality: for each coping strategy, the field of study and all its interactions with the predictor, the moderators and the predictor × moderator terms were entered as a block, and the significance of the resulting increase in explained variance (ΔR²) was evaluated with an F-test.

## Results

The data collected from 607 university students provided a basis for evaluating the interplay between perceived stress, coping strategies, and psychological resilience. Consistent with the transactional model of stress discussed above, our analysis examines not only the underlying intensity of academic stress but also the internal resources students draw upon to manage these demands.

### Analysis of stress levels, resilience, and coping strategies

The mean score for perceived stress (PSS-10) was 22.54 (SD = 7.04), which corresponds to a STEN score of 7.29 (SD = 1.99). This indicates that the respondents experienced moderate to elevated levels of stress. In terms of coping strategies, the most used were acceptance (M = 1.94; SD = 0.65) and active coping (M = 1.85; SD = 0.52). The least frequently used strategies were turning to religion (M = 0.62; SD = 0.93) and helplessness (M = 1.05; SD = 0.53). These average values indicate a moderate, functional approach to coping with difficulties, with a predominance of constructive and active responses.

Among personal and resilience resources, the highest scores were achieved in openness to new experiences and sense of humor (M = 2.72; SD = 0.68) and tolerance for failure and treating life as a challenge (M = 2.50; SD = 0.71). The lowest, though still significant, scores were in positive attitude (M = 1.93; SD = 0.81). The total resilience level (SPP-25) averaged 59.39. This indicates a moderately high level of psychological resilience in the entire group. At the same time, the large variation in results (SD = 15.37) suggests significant individual differences in participants’ available psychological resources.

The results obtained by respondents on the Perceived Stress Scale (PSS-10), Resilience Scale (SPP-25), and Mini-COPE Stress Coping Inventory are summarized in Table 2. We assessed internal consistency reliability for all measurement scales using Cronbach’s alpha coefficient. The obtained values indicated satisfactory reliability for all instruments, with all scales exceeding the commonly accepted threshold of α > 0.70, confirming good internal consistency.

**Table 2:** Descriptive statistics for PSS-10, SPP-25, and Mini-COPE scores.

Variables	Cronbach’s alpha	Mean (SD)	Median (min-max)	Skewness	Kurtosis
**Stress level (PSS-10)**					
Level of perceived stress	0.861	22.54 (7.04)	23.00 (2-38)	−0.33	−0.49
**Resilience level (SPP-25)**					
Resilience level (overall score)	0.823	59.39 (15.37)	61.00 (6-100)	−0.33	0.10
Perseverance and determination in action	0.887	2.41 (0.79)	2.40 (0.2-4)	−0.39	−0.17
Openness to new experiences and a sense of humor	0.784	2.72 (0.68)	2.80 (0-4)	−0.59	0.36
Personal comptetence to cope and tolerance of negative emotions	0.832	2.32 (0.79)	2.40 (0-4)	−0.40	−0.16
Tolerance for failure and treating life as a challenge	0.922	2.50 (0.71)	2.60 (0.2-4)	−0.49	0.19
An optimistic outlook on life and the ability to mobilize in difficult situations	0.845	1.93 (0.81)	2.00 (0-4)	−0.05	−0.39
**Coping strategies (Mini-COPE)**					
Active coping	0.823	1.85 (0.52)	1.83 (0-3)	−0.23	0.28
Helplessness	0.788	1.05 (0.53)	1.00 (0-3)	0.48	0.22
Support seeking	0.843	1.80 (0.77)	2.00 (0-3)	−0.39	−0.50
Avoidant behavior	0.811	1.40 (0.46)	1.33 (0-3)	0.14	0.22
Turn to religion	0.766	0.62 (0.93)	0.00 (0-3)	1.30	0.41
Acceptance	0.833	1.94 (0.65)	2.00 (0-3)	−0.49	0.30
Sense of humor	0.803	1.19 (0.72)	1.00 (0-3)	0.35	−0.22

*Note: PSS-10 – Perceived Stress Scale; SPP-25 – Resilience Scale; SD – standard deviation; min – minimum; max – maximum.*

To test whether the medical and non-medical student subgroups were homogeneous, we conducted post hoc comparisons between groups to examine potential differences. The analyses were performed using independent-samples t-tests (for variables with approximately normal distributions) and Mann–Whitney U tests (for variables that did not meet the assumption of normality). Additionally, we compared moderating models between groups by analyzing differences in regression parameters.

The results showed no statistically significant differences between medical and non-medical students in any analyzed variable or in the structure of the moderating relationships. Therefore, the field of study did not significantly differentiate the relationships examined in the present sample, and the two groups could be combined.

Detailed results of these comparisons are presented in Table 3. Medical students (n = 374) and non-medical students (n = 233) did not differ significantly in perceived stress (t(605) = 0.22; p = 0.826; d = 0.02), in the overall level of resilience (t(605) = 1.46; p = 0.146; d = 0.12) or in any of its five components (all p ≥ 0.335; |d| ≤ 0.08), nor in any of the seven coping strategies (all p ≥ 0.161; |r| ≤ 0.07). The smallest p-value obtained across the 14 comparisons (p = 0.146) remained well above the Bonferroni-adjusted threshold (α = 0.0036), and all effect sizes were negligible. The comparison of the moderation models between the two subgroups (Table 4) led to the same conclusion: for every coping strategy, adding the field of study together with all its interaction terms produced a non-significant increment in explained variance (ΔR² = 0.016–0.026; F(12, 583) = 0.86–1.37; all p ≥ 0.174).

**Table 3:** Comparison of medical and non-medical students on perceived stress, resilience and coping strategies.

Variable	Medical students (n = 374) M (SD)	Non-medical students (n = 233) M (SD)	Test statistic	p	Effect size
**Stress level (PSS-10)**					
Level of perceived stress	22.59 (7.29)	22.46 (6.64)	t(605) = 0.22	0.826	d = 0.02
**Resilience level (SPP-25)**					
Resilience level (overall score)	60.11 (15.58)	58.24 (14.98)	t(605) = 1.46	0.146	d = 0.12
Perseverance and determination in action	2.42 (0.77)	2.39 (0.83)	t(605) = 0.38	0.705	d = 0.03
Openness to new experiences and a sense of humor	2.73 (0.69)	2.71 (0.66)	t(605) = 0.26	0.796	d = 0.02
Personal competence to cope and tolerance of negative emotions	2.34 (0.77)	2.29 (0.82)	t(605) = 0.80	0.422	d = 0.07
Tolerance for failure and treating life as a challenge	2.48 (0.73)	2.54 (0.68)	t(605) = −0.96	0.335	d = −0.08
An optimistic outlook on life and the ability to mobilize in difficult situations	1.94 (0.82)	1.91 (0.79)	t(605) = 0.43	0.671	d = 0.04
**Coping strategies (Mini-COPE)**					
Active coping	1.84 (0.50)	1.86 (0.55)	U = 42026; Z = −0.74	0.462	r = −0.04
Helplessness	1.07 (0.52)	1.02 (0.55)	U = 46514; Z = 1.40	0.161	r = 0.07
Support seeking	1.78 (0.77)	1.83 (0.77)	U = 42102; Z = −0.70	0.485	r = −0.03
Avoidant behavior	1.39 (0.46)	1.42 (0.45)	U = 42526; Z = −0.50	0.619	r = −0.02
Turn to religion	0.64 (0.94)	0.58 (0.91)	U = 44456; Z = 0.42	0.674	r = 0.02
Acceptance	1.93 (0.65)	1.95 (0.64)	U = 42893; Z = −0.32	0.747	r = −0.02
Sense of humor	1.20 (0.72)	1.17 (0.73)	U = 44744; Z = 0.56	0.577	r = 0.03

*Note. M – mean; SD – standard deviation. t – independent-samples t-test (variables with approximately normal distributions); U – Mann–Whitney U test with the standardised Z statistic (variables that did not meet the assumption of normality). Effect sizes: d – Cohen’s d; r – rank-biserial correlation. Bonferroni-adjusted significance threshold for 14 comparisons: α = 0.0036.*

**Table 4:** Comparison of the moderation models between medical and non-medical students.

Coping trategy (predictor)	ΔR^2^	F (12, 583)	p
Active coping	0.020	1.06	0.391
Helplessness	0.026	1.37	0.174
Support seeking	0.016	0.86	0.591
Avoidant behavior	0.023	1.19	0.285
Turn to religion	0.024	1.27	0.229
Acceptance	0.017	0.89	0.553
Sense of humor	0.025	1.30	0.217

*Note. For each coping strategy, the reduced model contained the strategy, the five resilience resources and their strategy × resource interactions; the full model additionally contained the field of study (medical vs. non-medical) and all of its interactions with these terms (12 df). ΔR² – increment in explained variance attributable to the field of study; F – F-test of the added block. None of the increments reached statistical significance.*

### Moderation analysis

The moderation analysis examined whether selected personal resources moderate the relationship between coping strategies and perceived stress (Table 5). The analysis included seven coping strategies. Multicollinearity diagnostics indicated no substantial collinearity among predictors. Variance Inflation Factor (VIF) values ranged from 1.08 to 1.74, while tolerance values ranged from 0.57 to 0.93, remaining well within recommended thresholds. Assessment of homoscedasticity using plots of standardized residuals versus predicted values revealed no systematic patterns, suggesting that the assumption of constant error variance was satisfied. Additionally, the Breusch–Pagan test was non-significant (χ² = 6.12, p = 0.41). Residual diagnostics indicated an approximately normal distribution of residuals. Visual inspection of Q–Q plots showed only minor deviations from normality. The Shapiro–Wilk test was non-significant (W = 0.987, p = 0.18). Standardized residuals ranged from −2.74 to 2.61, with no values exceeding the critical threshold of ± 3.0. Cook’s distance values ranged from 0.001 to 0.09, indicating no influential observations.

**Table 5:** Analysis of personal resource moderation with coping strategies and experienced stress.

Strategy	Moderator	R	R²	p	coeff	R²-chng	p
Active coping	Perseverance and determination	0.2229	0.0497	0.0185	0.0026	0.0005	0.7426
	Openness and humor	0.2576	0.0664	0.0037	−0.0013	0.0002	0.8505
	Coping skills and emotional tolerance	0.2486	0.0618	0.0058	0.0109	0.0108	0.1351
	Failure tolerance and challenge view	0.2423	0.0587	0.0078	0.0111	0.0096	0.1585
	Optimism and self‑motivation.	0.2219	0.0493	0.0192	−0.0007	0.0000	0.9308
Helplessness	Perseverance and determination	0.1112	0.0124	0.4851	−0.0050	0.0018	0.5495
	Openness and humor	0.1074	0.0115	0.5164	0.0050	0.0023	0.5034
	Coping skills and emotional tolerance	0.0909	0.0083	0.6524	0.0071	0.0043	0.3588
	Failure tolerance and challenge view	0.1542	0.0238	0.1926	−0.0118	0.0103	0.1511
	Optimism and self‑motivation.	0.0983	0.0097	0.5914	0.0070	0.0039	0.3812
Seeking support	Perseverance and determination	0.3037	0.0922	<0.001	−0.0056	0.0025	0.4595
	Openness and humor	0.3031	0.0919	<0.001	−0.0050	0.0025	0.4626
	Coping skills and emotional tolerance	0.3006	0.0904	<0.001	−0.0002	0.0000	0.9774
	Failure tolerance and challenge view	0.3107	0.0965	<0.001	0.0081	0.0054	0.2794
	Optimism and self‑motivation.	0.3040	0.0924	<0.001	0.0030	0.0008	0.6793
Avoidant behavior	Perseverance and determination	0.2468	0.0609	0.0063	−0.0002	0.0000	0.9808
	Openness and humor	0.2630	0.0692	0.0028	0.0100	0.0095	0.1585
	Coping skills and emotional tolerance	0.2549	0.0650	0.0042	0.0082	0.0061	0.2612
	Failure tolerance and challenge view	0.2577	0.0664	0.0037	0.0093	0.0067	0.2377
	Optimism and self‑motivation.	0.2483	0.0616	0.0059	0.0053	0.0023	0.4901
Turn to religion	Perseverance and determination	0.0962	0.0093	0.6087	−0.0096	0.0075	0.2232
	Openness and humor	0.0695	0.0048	0.8131	0.0048	0.0024	0.4946
	Coping skills and emotional tolerance	0.1148	0.0132	0.4563	0.0010	0.0001	0.8907
	Failure tolerance and challenge view	0.1607	0.0258	0.1616	−0.0170	0.0240	**0.0291**
	Optimism and self‑motivation.	0.1110	0.0123	0.4872	−0.0087	0.0067	0.2493
Acceptance	Perseverance and determination	0.2015	0.0406	0.0431	−0.0014	0.0001	0.8619
	Openness and humor	0.2145	0.0460	0.0260	−0.0045	0.0020	0.5178
	Coping skills and emotional tolerance	0.2456	0.0603	0.0067	−0.0128	0.0154	0.0744
	Failure tolerance and challenge view	0.2090	0.0437	0.0325	−0.0062	0.0032	0.4220
	Optimism and self‑motivation.	0.2085	0.0435	0.0331	0.0012	0.0001	0.8731
Sense of humor	Perseverance and determination	0.1005	0.0101	0.5732	−0.0011	0.0001	0.8898
	Openness and humor	0.0724	0.0052	0.7931	−0.0034	0.0011	0.6435
	Coping skills and emotional tolerance	0.1508	0.0228	0.2103	−0.0137	0.0171	0.0659
	Failure tolerance and challenge view	0.0862	0.0074	0.6905	−0.0019	0.0003	0.8143
	Optimism and self‑motivation.	0.0945	0.0089	0.6234	−0.0051	0.0021	0.5156

The results show that most models demonstrate a significant relationship between the strategy and the main outcome (p < 0.05), but interaction effects, i.e., the role of moderators, rarely reach statistical significance. R values ranged from 0.07 to 0.31, indicating weak to moderate relationships. Only the interaction between the “turn to religion” strategy and the “tolerance for failure” moderator was significant (p = 0.0291), which, in our cross-sectional data, suggests a statistically significant interaction rather than a demonstrated causal strengthening or weakening of this relationship. In several other cases, the moderation effect was close to significance, e.g., for “sense of humor” and “personal competence” (p = 0.0659).

## Discussion

The analysis of 607 students showed a co-occurrence of moderate to elevated perceived stress, relatively high psychological resilience, and a predominance of adaptive coping strategies, particularly active coping and acceptance. This pattern suggests that despite substantial academic demands, students report using generally functional coping resources. The sample was substantially overrepresented by medical students. This group, although exposed to chronic stress and high academic demands, is often characterized by high cognitive competence and a task-oriented coping style, which explains the mobilization of available mental resilience resources [20]. This may have contributed to this profile, although the cross-sectional design does not allow causal inference regarding this association.

Overall, the level of perceived stress observed in this study was comparable to findings from previous research among medical and non-medical student populations in Poland and other countries [21–23], with some variation across contexts. Although contextual factors such as study–work balance or perceived social support have been proposed to explain these differences, cross-sectional comparisons do not allow firm conclusions regarding their influence.

Psychological resilience in the present sample was moderately high, with particularly elevated scores in openness to experience and sense of humor. This means that the respondents have significant psychological resources that support effective functioning and adaptation to stress. These resources are expressed in a flexible approach, a sense of agency, optimism, and readiness to act. These findings are consistent with prior studies conducted among Polish healthcare-related, student populations [24]. The likely reason for this similarity is the overrepresentation of medical students in our study. Medical training prepares students to function in high-stress, uncertain, and time-pressured situations. The high level of resilience in both studies may also indicate that people who choose these fields of study already have a greater ability to adapt to difficult situations during their studies, or develop it during their education. High scores on openness to new experiences may reflect a willingness to learn, which is crucial in the dynamic, changing healthcare environment.

The results obtained, indicating more frequent use of adaptive strategies, are consistent with studies conducted among students in Poland and other European countries, where these strategies are among the most frequently reported ways of coping with academic stress [7,25,26]. Research using tools based on the COPE concept shows that students are less likely to use passive strategies such as helplessness or denial, as reflected in the results obtained [27,28]. The low intensity of turning to religion is consistent with results from studies among students in cultural contexts with moderate or low religiosity, where this strategy is among the least frequently chosen for everyday coping with academic difficulties [7,29]. Thus, the distribution of coping strategy use aligns with the pattern observed in the literature: a preference for active and cognitive strategies, with limited use of passive and religious strategies among the student population.

The moderation analysis results indicate that although coping strategies and personal resources remain significantly related to perceived stress levels, their interaction rarely yields significant moderating effects. This is consistent with reports from other cross-sectional studies, in which interaction effects between psychological variables are usually weaker and harder to detect than the main effects [30,31]. A significant moderating effect was found only for the “turn to religion” strategy and tolerance for failure, which may suggest that this strategy is secondary and that its significance emerges primarily in specific configurations of personal resources. Similar conclusions appear in the literature, which emphasizes that turning to religion is not a universal strategy but is used selectively, especially in situations of reduced sense of control or limited mental resources [32,33]. Moderating effects of sense of humor and personal competence that are close to statistical significance may indicate a potential, albeit weak, interaction with coping strategies, warranting further longitudinal research or larger sample sizes. At the same time, the study group’s high overall psychological resilience may have masked the moderating effects, limiting variability in the results and thus reducing the strength of the observed interactions. Overall, the results suggest that personal resources primarily function as independent protective factors, while their role as moderators of the relationship between coping strategies and stress is limited.

The study has several limitations. Its cross-sectional design precludes causal inference regarding the observed relationships. The sample was also not fully representative of the general student population due to a predominance of women and medical students, which may limit generalizability. In addition, data were collected through an online CAWI survey, which may have introduced self-selection bias related to interest in mental health or online engagement. Variability in data collection timing across the academic year may also have influenced reported stress levels. Finally, the lack of stratification by field of study limited the ability to examine potential subgroup differences, and future research should consider longitudinal designs and alternative analytical approaches, including mediation models, to better capture underlying mechanisms.

## Conclusions

The study shows that, in this sample, moderate to high stress co-occurs with relatively strong adaptive resources, as reflected in resilience scores and the predominance of active coping strategies. The coexistence of high tension with relatively high mental resilience and the dominance of active strategies (such as acceptance and action) suggests that students have developed the ability to mobilize resources in the face of academic demands. In this group, resilience appears to be an independent, fundamental resource associated with lower levels of perceived stress. Our cross-sectional analysis shows a stronger direct association with stress than consistent moderating effects on individual coping strategies. Therefore, any casual interpretations discussed here should be treated as hypotheses for future longitudinal research.

These results have significant implications for designing psychological support in academic settings. Given that it is impossible to eliminate stressors in demanding fields of study, our findings suggest that educational and intervention programs could strengthen existing resilience resources and promote a task-oriented coping style. Future longitudinal or intervention studies are needed to verify whether such approaches effectively reduce academic stress. Investing in the development of personal competencies, such as openness or the ability to distance oneself (humor), can be a key element in preventing academic burnout and preparing students for their future careers.

## Supporting information

S1 Dataset An anonymized dataset containing responses from 607 participants was used in the statistical analyses. (XLSX)
